# Correlation of Pelvic Organ Prolapse Diagnoses with Google Trends in the Flint Metropolitan Area

**DOI:** 10.51894/001c.143934

**Published:** 2025-09-30

**Authors:** Marcie Bingham

**Affiliations:** 1 Henry Ford Genesys

## 22

### INTRODUCTION

Pelvic organ prolapse while a benign condition can have significant quality of life implications including difficulty with voiding or defecating, uncomfortable sensations, or difficulty with intercourse. The ability to predict health care utilization using Google Trends could be a helpful tool in allowing offices to better allocate their clinic schedules, operating room time, and other resources to meet predictable increases in need in the population. Other studies looking at osteoarthritis and influenza have shown that Google Trends is a resource that can accurately predict diagnoses by time and location.

### OBJECTIVES

This study was designed to compare Google Trends search data in the Flint Metropolitan Area with local diagnosis rates of pelvic organ prolapse to determine if there was a statistically significant correlation.

### METHODS

This retrospective study looked at publicly accessible search data from Google Trends in the year 2022 by month and compared ICD N81 codes billed per month at Ascension Flint OBGYN Clinic, Women’s Specialty Associates, Women’s Integrated Health, and Ascension Medical Group OBGYN to Google search trend rates per month of the terms “vaginal bulge”, “pelvic floor”, “bladder drop”, “prolapse”, and “cystocele”.

### RESULTS

There were a total of 518 diagnoses of pelvic organ prolapse between Ascension Flint OBGYN Clinic, Women’s Specialty Associates, Women’s Integrated Health, and Ascension Medical Group OBGYN. January had the peak interest of search terms being “vaginal bulge” to which all other months and search terms were compared to. A linear regression was performed that showed a correlation with a p-value of 0.077 and R value of 0.53.

### CONCLUSION

While the data was limited as some months had no Google search results at all and there was an overall small sample size the results show promise that perhaps there could be a correlation with Google search trends and incidence of pelvic organ prolapse.

